# The Role of L-Arginine and Liposomal Vitamin C Supplementation as an Adjunct in Seasonal Respiratory Viral Infection Recovery

**DOI:** 10.3390/arm94010011

**Published:** 2026-02-09

**Authors:** Valentina Trimarco, Paola Gallo, Seyedali Ghazihosseini, Alessia Izzo, Paola Ida Rozza, Alessandra Spinelli, Stefano Cristiano, Carlo De Rosa, Felicia Rozza, Carmine Morisco

**Affiliations:** 1 Department of Neuroscience, Reproductive Sciences, and Dentistry, “Federico II” University, 80131 Naples, Italy; valentina.trimarco@unina.it; 2 Department of Advanced Biomedical Sciences, “Federico II” University, 80131 Naples, Italy; paola.gallo@unina.it (P.G.); ali_ghz_95@yahoo.com (S.G.); alessiaizzo45@gmail.com (A.I.); alessandra.spinelli@unina.it (A.S.); stefano.cristiano@unina.it (S.C.); 3 Unicamillus International Medical University, 00199 Rome, Italy; paolarozza21@gmail.com (P.I.R.); felicia_rozza@libero.it (F.R.); 4 Legal Medicine University of Tuscia, 1100 Viterbo, Italy; derosa.medicolegale@gmail.com

**Keywords:** endothelial dysfunction, human coronavirus, human respiratory syncytial virus, influenza virus, parainfluenza virus, oxidative stress, rhinovirus

## Abstract

**Highlights:**

**What are the main findings?**

- Many respiratory viruses are able to induce endothelial dysfunction.
- L-Arginine and Vitamin C interfere with the viral-induced inflammatory response

**What are the implications of the main findings?**

- The combination of L-Arginine and Vitamin C seems to represent a nutritional support able to relieve the clinical symptoms of respiratory seasonal viral infection.

**Abstract:**

Respiratory seasonal viral infections remain one of the most important issues in community medicine. The heterogeneity of etiological agents and the characteristics of the hosts airway antiviral defenses account for the complex management of these infections. The clinical consequence of this picture is that, despite the widespread use of vaccination as the primary prevention strategy, the rates of acute respiratory complications remain still high. In addition, they determine post-infectious fatigue and organ dysfunction. Inflammation and oxidative stress are the principal pathogenic mechanisms responsible for clinical complications during respiratory seasonal viral infections. Nowadays, a growing body of evidence indicates that adjunctive nutritional support can contribute to relieve the symptoms during the acute and subacute phases of respiratory viral infections. We assess the data in the literature regarding the combination of L-Arginine and Liposomal Vitamin C as adjuvant treatment for respiratory seasonal viral infections. The database of the National Library of Medicine (PubMed) was searched using the keywords “L-Arginine, Vitamin C, dietary supplements, seasonal respiratory viral infections”. The treatment of symptoms during acute and post-acute respiratory viral infections requires an integrated approach that includes vitamins and nutritional supplementation. The combination of L-Arginine and Liposomal Vitamin C seems to represent a nutritional support able to mitigate symptoms occurring during the acute or post-acute phase of infection.

## 1. Introduction

The management of seasonal respiratory viral infections remains one of the most important issues of community medicine, in terms of prevention as well as of treatment of symptoms and sequels. The first issue with respiratory seasonal viral infections is the extreme heterogeneity of etiological agents, which include the influenza virus, the human coronavirus, the human respiratory syncytial virus, the parainfluenza virus, and the rhinovirus [1]. Furthermore, another aspect that significantly contributes to the worsening of the scenario is the heterogeneity in the host’s airway antiviral defenses. These include the mucosal surface of the respiratory epithelium, the innate immune responses, and the virus-specific adaptive immunity [1]. The consequence of this multifaceted picture is that despite the widespread use of vaccination as primary prevention strategy in most frail individuals, the rates of acute respiratory complications still remain high. In fact, it has been estimated that lower-respiratory-tract diseases account for 5,7 million hospitalizations worldwide, with almost 300,000 deaths every year [2]. Hospitalizations are more frequent in people aged 65 years and over and in children younger than 5 years [3].

The clinical manifestations and complications of seasonal respiratory viral infections range from asymptomatic infections without fever to acute respiratory distress syndrome. However, cardiac, gastrointestinal, renal, musculoskeletal, and neurological manifestations [4] are also described. In addition, often they determine post-infectious fatigue and organ dysfunction, due to inflammation and oxidative stress.

The aim of this communication was to assess the data regarding the combination of L-Arginine and Liposomal Vitamin C as adjuvant treatment of respiratory seasonal viral infections. For this purpose, the database of the National Library of Medicine (PubMed) was searched using the following keywords: “L-Arginine, Vitamin C, dietary supplements, seasonal respiratory viral infections”.

The treatment of respiratory viral infections is based on the administration of viral neuraminidase inhibitors and viral polymerase inhibitors. These antiviral treatments are aimed at inhibiting the viral replication in infected cells. In addition, anti-inflammatory medications are used. However, their efficacy in this context is not proven yet or still limited. For instance, colchicine has been proposed as an anti-inflammatory agent to relieve the symptoms of viral seasonal infections including COVID-19. However, since it has not exhibited significant benefit in reducing mortality or severity in major trials and is associated with considerable gastrointestinal side effects [5], colchicine is not currently a recommended treatment for the routine management of post-acute viral syndromes.

Interestingly, nowadays, a growing body of evidence indicates that adjunctive nutritional support can contribute to the relief of symptoms during the acute and subacute phases of respiratory viral infections. In particular, the combination of L-Arginine and Liposomal Vitamin C has been proposed as a nutritional support to mitigate symptoms existing during the acute or post-acute phase of infection. L-Arginine, a semi-essential amino acid, is involved in several biological processes, but a crucial role remains for endothelial function. It is the substrate of endothelial nitric oxide (NO) synthase (eNOS), a key enzyme for NO synthesis, involved not only in the control of vascular tone and thrombosis but also in the modulation of the immune response [6]. In fact, the immune system depends on the amount of L-Arginine available in the body. Moreover, it has been reported that the T cell function depends on L-Arginine levels [7], and it has been documented that L-Arginine is required for the macrophage M1-to-M2 switch [8]. Definitely, it is reasonable to assert that L-arginine plays a key role in the immune homeostasis. In addition, Vitamin C (Ascorbic Acid) is a potent antioxidant and an essential co-factor for numerous biological processes, it significantly contributes to the regulation of immune defense, particularly in T-cells [9]. Their synergistic action interferes with the molecular mechanisms evoked by inflammation. Thus, it is reasonable to speculate that this action can be translated, in clinical practice, to the relief of symptoms occurring during respiratory viral infections.

## 2. The Lesson from SARS-CoV-2 Infection

The coronavirus disease 2019 (COVID-19) pandemic highlighted the need for deeper investigations into potential therapies or prophylaxis against seasonal respiratory viral infections and can be considered as the paradigm of these diseases. Experimental evidence indicates that the endothelium is a target of the SARS-CoV-2 infection, and endothelial dysfunction plays a key role in the genesis of symptoms of acute and chronic COVID-19 (Long COVID) [10]. On the other hand, there is convincing evidence that the amelioration of endothelial dysfunction was associated with improvements in clinical outcome [11,12,13].

A large-scale survey (The LINCOLN Survey) [14], and subsequent clinical trials have investigated the effects of a combined L-Arginine (to improve endothelial function) and Liposomal Vitamin C (to reduce oxidation) supplementation (similar to the formulation in Bioarginina C) in patients suffering from Long COVID [15]. Moreover, in this context, it was revealed that L-Arginine significantly reduced the circulating levels of pro-inflammatory IL-2, IL-6, and IFN-γ and increased the levels of the anti-inflammatory IL-10 [16]. The results of these investigations indicated a significant reduction in debilitating symptoms like fatigue (asthenia) and shortness of breath (dyspnea), as well as an improvement in physical performance. The results of this study highlight the mechanistic link between the bio-markers of inflammation and the relief of symptoms, suggesting a potential role for this specific combination in accelerating the recovery phase following severe viral-induced stress. stress Therefore, the stabilization of the endothelial barrier by reducing its permeability could be useful to reduce the clinical symptoms occurring during viral infections, even if the principal target of the viral infection is the respiratory tract. This capability represents the rationale for the use of the combination of L-Arginine and Vitamin C in respiratory viral infections beyond SARS-CoV-2. In fact, although seasonal respiratory viral infections have different etiologic agents, they share similar risk factors and pathogenic mechanisms responsible for the clinical outcome [17]. In addition, they have similar clinical presentations [18]. It has been reported that infections induced by influenza viruses can persist for the long term with malaise and cough [18]. These data highlight the need to include, in the therapy of these infections, principles that interfere with the pathogenic mechanisms of target organ damage beyond the etiological agent.

The usefulness of diet patterns, dietary supplements, and healthy behavior to reduce the symptoms of acute and post-acute phases of COVID-19 infection has been clearly documented. In particular, in Egyptian students, it was observed that those were non-obese, non-smokers, physically trained, and who took vitamins, herbal supplements, and ate a healthy diet had a strong anti-SARS-CoV-2 humoral immune response [19]. Interestingly, it has been reported that adherence to the Mediterranean diet is able to reduce the risk of respiratory infections including SARS-CoV-2. The beneficial effects of the Mediterranean diet are mainly mediated by the low levels of saturated fats, that, in turn, account for the decrease in pro-inflammatory cytokines [20]. In addition, it has also been documented that vitamin supplementation exerts a beneficial effect on the clinical outcome of SARS-CoV-2 infections. In particular, an inverse relationship has been reported between the levels of Vitamin D and the severity of SARS-CoV-2 infection. Thus, Vitamin D supplementation has been considered an efficient co-treatment for COVID-19 [21]. Similarly, it has been reported that, during SARS-CoV-2 infection, the supplementation of Vitamin A reduces fever, fatigue, and pain. The antioxidant effect of vitamin C is well recognized. Moreover, vitamin C is also an important immunomodulator acting on T-lymphocyte maturation, phagocytosis, and chemotaxis. For this reason, vitamin C is successfully used in the prevention of complications of sepsis and pneumonia. The beneficial effects of vitamin C on respiratory function have been also documented during the COVID-19 pandemic. In a randomized trial conducted on patients with severe COVID-19 infection, it has been reported that high doses of vitamin C had beneficial effects on the symptoms and duration of hospitalization [22]. It must be underlined that the supplementation role of Vitamin C as an adjuvant of treatment of COVID-19 and other viral respiratory infections is still debated. Several determinants can account for these doubts, such as the different doses and preparations of vitamin C supplements used in the studies, as well as the differences in the designs, in the sample size, and in the clinical characteristics of the patients enrolled in trials. To ameliorate the bioavailability, the majority of studies in COVID-19 infection tested high doses of intravenous administration of vitamin C [23]. Interestingly, in the LINCOLN survey [14], they used a liposomal preparation of vitamin C to enhance the bioavailability of the oral administration of vitamin C.

The lesson coming from the COVID-19 pandemic is that the treatment of symptoms during acute and post-acute respiratory viral infections requires an integrated approach that includes vitamins and nutritional supplementation.

## 3. Endothelial Dysfunction and Viral Infections

The link between viral infections and endothelial dysfunction was unknown before the SARS-CoV-2 pandemic. Now, it is clear that beyond SARS-CoV-2, respiratory syncytial viruses and influenza A (H1N1) viruses are able to induce endothelial dysfunction. Since endothelium expresses many co-factors required for the internalization of several viruses, it can be considered as a target organ of viral infections, including seasonal respiratory infections. This phenomenon is not negligible, since it can cause the transition from local to systemic inflammation.

**Figure 1:**
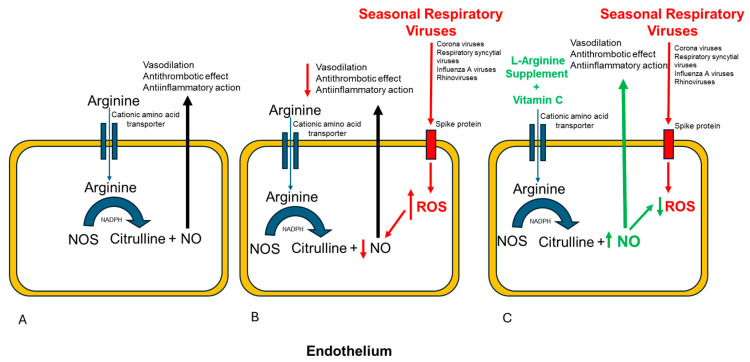
Role of L-Arginine and Vitamin C on endothelial function in seasonal viral respiratory infections. (**A**) In physiological conditions, in endothelial cells, the endothelial nitric oxide synthase (NOS) catalyzes the transformation of arginine to nitric oxide (NO) and citrulline. This is an NADPH-dependent reaction. NO plays a key role in vascular homeostasis through a vasodilatory action, with antithrombotic and anti-inflammatory effects. (**B**) Endothelium is a target organ of seasonal respiratory viruses. The viruses, through their spike proteins localized on cell membrane, are translocated into the cytosol. Here, they stimulate the production of reactive species of oxygen (ROS), which, in turn interfere with the stability and production of NO, resulting in endothelial dysfunction. (**C**) The supplementation of Arginine plus Vitamin C, antagonizes the oxidative stress evoked by the excess of ROS and, at the same time, enhances the substrate of NOS, resulting in restoration of NO availability and endothelial function.

The pathogenic mechanism that accounts for the development of endothelial dysfunction during respiratory viral infections is oxidative stress. The inflammatory cells are involved in this phenomenon through the direct generation of reactive oxygen species (ROS) or through the release of inflammatory mediators [24]. The generation of ROS molecular mechanisms during viral infections is a complex and multifactorial process that involves in immune cells the dysregulation of NADPH oxidase and xanthine oxidase, and in neutrophils the release of myeloperoxidase, as well as in non-immune cells the functional abnormalities of mitochondria, peroxisomes, and endoplasmic reticulum associated cytochrome oxidoreductase [24]. In response to the inflammatory activation, endothelial cells produce enzymes that account for the glycocalyx detachment, leading to disruption of endothelial barrier function resulting in an increase in vascular permeability. This represents the pathogenic mechanism of airway obstruction and lung injury [25]. Thus, endothelial damage represents the key point that accounts for the clinical evolution of respiratory viral infections. The beneficial effects of L-Arginine on endothelial function have been reported [26], providing a strong rationale for its use in patients with respiratory viral infections. (Figure 1).

## 4. Rationale for Use of Antioxidants

Several data report the development of oxidative stress in different viral infections including seasonal respiratory diseases [27]. During influenza, respiratory syncytial virus, rhinovirus, and corona virus infections, an excessive amount of ROS is produced in several tissues and organs such as alveolar epithelium and endothelium [28]. Since oxidative stress is the common denominator of target organ damage during the seasonal respiratory viral infections, it is reasonable to assume that the use of antioxidants such as vitamins E, C, SH group donors (e.g., N-acetyl cysteine), iron chelating agents (deferoxamine), or activators of NRF2-driven gene expression could be useful in reducing the expression/release of pro-inflammatory mediators and help patients to recover from respiratory viral infections [29,30]. Moreover, decreasing oxidative stress by antioxidants may result in a reduced viral load [28]. Therefore, targeting oxidative stress plays a key role in the clinical management of seasonal respiratory viral infections. In this context, it has been reported that, in patients with COVID-19, high-doses of vitamin C have a beneficial effect on clinical outcomes of disease without adverse effects [23]. In addition, vitamin C is able to also modulate the viral-induced inflammatory response, through a reduction in the chemotaxis of neutrophils and lymphocytes and by interfering with the pro-inflammatory cytokine cascade, helping to alleviate the respiratory symptoms occurring during viral seasonal respiratory infections [9,31].

Moreover, the nutritional supplementation with L-Arginine plus Vitamin C has demonstrated the restoration of the bioavailability of NO in patients with Long Covid [32], suggesting that this supplement may be proposed to relieve the clinical symptoms of respiratory viral infections.

## 5. Conclusions

Although vaccination remains the undisputed cornerstone of public health defense against seasonal respiratory viral infections, the current data provide a compelling mechanistic and clinical rationale for the use of L-Arginine combined with Liposomal Vitamin C (as in Bioarginina^®^ C or other equivalent formulations) as an adjunctive nutritional strategy in the post-acute phase of systemic viral infections. Further randomized controlled clinical trials specifically targeting the recovery phase of seasonal influenza and comparing different L-Arginine/Vitamin C formulations are warranted to solidify these findings and to establish this as a standard post-vaccination and post-infection nutritional protocol.

## Data Availability

No new data were created or analyzed in this study. Data sharing is not applicable to this article.
